# Electrochemotherapy Effectiveness Loss due to Electric Field Indentation between Needle Electrodes: A Numerical Study

**DOI:** 10.1155/2018/6024635

**Published:** 2018-07-02

**Authors:** José Alvim Berkenbrock, Rafaela Grecco Machado, Daniela Ota Hisayasu Suzuki

**Affiliations:** ^1^Department of Electrical and Computer Engineering, University of Saskatchewan, Saskatoon, Canada; ^2^Department of Electrical and Electronics Engineering, Institute of Biomedical Engineering, Federal University of Santa Catarina, Florianópolis, SC, Brazil; ^3^Department of Anatomy and Cell Biology, University of Saskatchewan, Saskatoon, SK, Canada

## Abstract

Electrochemotherapy is an anticancer treatment based on applying electric field pulses that reduce cell membrane selectivity, allowing chemotherapy drugs to enter the cells. In parallel to electrochemotherapy clinical tests, in silico experiments have helped scientists and clinicians to understand the electric field distribution through anatomically complex regions of the body. In particular, these in silico experiments allow clinicians to predict problems that may arise in treatment effectiveness. The current work presents a metastatic case of a mast cell tumor in a dog. In this specific treatment planning study, we show that using needle electrodes has a possible pitfall. The macroscopic consequence of the electroporation was assessed through a mathematical model of tissue electrical conductivity. Considering the electrical and geometrical characteristics of the case under study, we modeled an ellipsoidal tumor. Initial simulations were based on the European Standard Operating Procedures for electrochemotherapy suggestions, and then different electrodes' arrangements were evaluated. To avoid blind spots, multiple applications are usually required for large tumors, demanding electrode repositioning. An effective treatment electroporates all the tumor cells. Partially and slightly overlapping the areas increases the session's duration but also likely increases the treatment's effectiveness. It is worth noting that for a single application, the needles should not be placed close to the tumor's borders because effectiveness is highly likely to be lost.

## 1. Introduction

Electrochemotherapy is an anticancer treatment based on pulsed electric fields and chemotherapy drugs. The electric field reduces the cell membrane's selectivity, promoting the cell's intake of chemotherapy drugs [1–3]. This biophysical phenomenon of decreasing cell membrane selectivity through electric field imposition is called electropermeabilization. The most accepted theory to explain such permeabilization considers that pores are induced around the cell membrane [4]. This process is called electroporation and considers that the membrane permeabilization can be reversible or irreversible depending on the membrane's capability of resealing the pores after the removal of the electric field [2, 5].

The reversible or irreversible electroporation can lead to different treatment outcomes. Reversible electroporation facilitates the uptake of chemotherapy drugs (e.g., bleomycin and cisplatin) by the cells and the selective death of tumor cells [1, 3]. When this delivery method is used, the cytotoxicity of bleomycin increases 300–700 times [3]. However, irreversible electroporation induces membrane disruption and consequently indiscriminate cell death [2]. In this sense, the ability to achieve the right parameters for targeting tumor cells has imposed challenges. These challenges are mainly consequences of the anatomical complexity and nonhomogeneous structures of which our tissues, organs, and bodies are composed.

The electric field distribution in biological tissues has been studied for decades, and recent in silico experiments have taken advantage of years of bioelectrical impedance analysis [6, 7] and powerful processors. Through in silico experiments, several different scenarios can be run, which have allowed scientists and clinicians to understand and predict problems in treatment effectiveness. The clinical treatment of electrochemotherapy has been used in in silico studies for years. In this therapy approach, there are three basic electrode types: (I) two parallel plates, (II) needles in two parallel rows, and (III) needles in the vertices of a hexagon—like a honeycomb [1]. Examples of the close relationship between in silico experiments and electrochemotherapy are found in studies on how to insert the needle electrodes for deeply seeded tumors [8, 9], for nonsymmetrical tumors [10], and for large tumors on the skin's surface [11–13].

Many earlier in silico studies did not consider electroporation as a factor influencing membrane conductivity and assumed a constant tissue electrical conductivity [14–16]. However, more recent studies have demonstrated the importance of considering such an effect for cancer treatment planning [9, 17, 18]. In the present work, a case of a metastatic mast cell tumor in a dog is studied. Mast cell tumors, or mastocytomas, are common tumors in the skin of dogs, and many of them are prone to local recurrence and metastasis [19]. We started this report with a specific treatment planning study to demonstrate the potential for efficiency loss when needle electrodes are used.

## 2. Materials and Methods

### 2.1. *In Vivo* Diagnosis

The patient was a 3-year-old male pitbull mixed-breed dog, 32 kg, with spontaneous nodular formations on the right posterior limb. The samples were collected from the right inguinal lymph node and were stained with May–Grünwald–Giemsa (MGG) dye for a histopathology examination. The patient was diagnosed with a metastatic mast cell tumor, and surgical removal was recommended.

**Figure 1:**
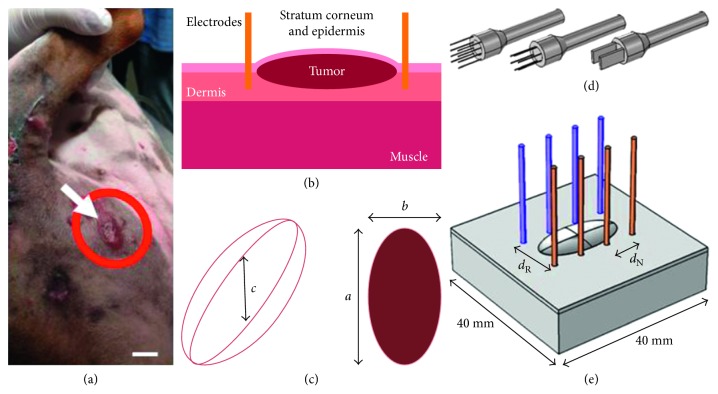
Schematic for modeling the tumor under study. From left to right, the target tumor, the geometrical parameters extraction and anatomical characterization, and the 3D insertion into the simulation environment. (a) A mast cell tumor in a 3-year-old male dog. The arrow indicates the modeled tumor. Scale: 10 mm. (b) Skin with three layers (*stratum corneum* with epidermis, dermis, and muscle), the tumor, and two representative electrode needles. (c) Approximated geometry and dimensional parameters of the tumor. (d) The three types of tested electrodes. (e) The 3D geometry model under study. The ellipsoid represents the tumor seeded on the skin layers, and the cylinders are the electrode needles.

The electrochemotherapy treatment was suggested as a potentially curative treatment option, and the patient was forwarded to the veterinary clinic that collaborated with this study. In Figure 1, the tumor chosen to be modeled is indicated with the arrow. This tumor was chosen because of its expressiveness rather than the others. The tumor dimensions were 20 mm along its longest diameter and 10 mm on the other superficial diameter (orthogonal axis).

### 2.2. In Silico Modeling

#### 2.2.1. Geometry and Tissue Properties

The data were made available by the clinic and patient owner. The tumor under study (Figure 1) was 3D modeled in the simulation environment (Figure 1), considering the parameters shown in the Figures 1 and 1. The tumor had its shape approximated to an ellipsoidal mass, with *a*, *b*, and *c* equal to 20 mm, 10 mm, and 1.25 mm, respectively (Figure 1). The two orthogonal surface diameters were *a* and *b*. The tumor depth *c* was estimated through the following equation [1]:

(1) $$\begin{array}{c} \text{Vol}=\frac{4}{3}\pi abc=\frac{\pi }{6}ab^{2}. \end{array}$$

The skin tissue was modeled with a surface area of 40 × 40 mm, and it was divided into three different layers. The deepest layer was the muscle with 10 mm of thickness; above it, was the dermis layer with 1 mm, followed by the *stratum corneum* (*SC*) and epidermis layer with 0.05 mm of thickness (Figure 1). The distance between the anode and the cathode for the parallel rows (*D*_R_) was 10 mm. The needles in the same row were (*D*_N_) 7 mm apart, and their radius was 0.64 mm.

**Figure 2:**
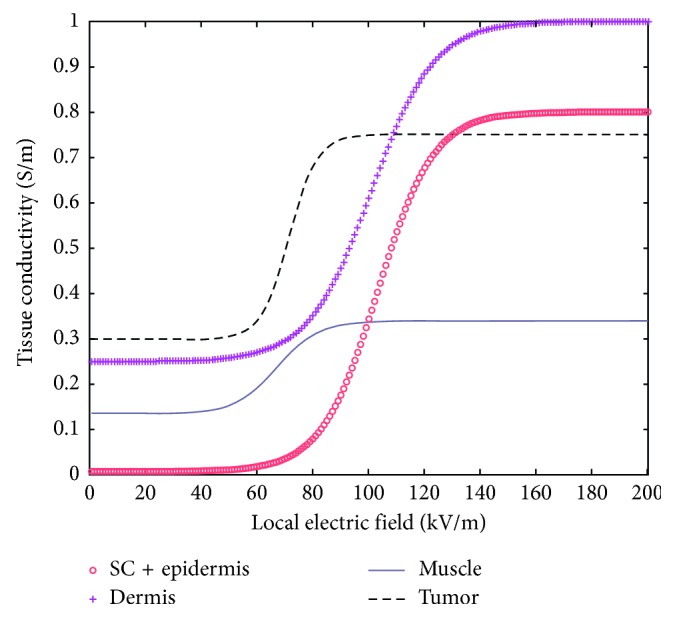
Curves for tissue conductivity dependent on the local electric field.

All tissues were considered homogeneous, and the electrical conductivity assigned to each skin layer and tumor is listed in the first column of Table 1. The macroscopic consequence of the electroporation is the increase in electrical conductance. Such behavior may be represented by the following mathematical model with a sigmoid shape (Figure 2) [20]:

(2) $$\begin{array}{c} \sigma \left(E\right)=\sigma_{0}+\frac{\sigma_{\max}-\sigma_{0}}{1+10\cdot e^{-\left(E-A/B\right)}}, \end{array}$$

(3) $$\begin{array}{c} A=\frac{E_{\text{irrev}}+E_{\text{rev}}}{2}, \end{array}$$

(4) $$\begin{array}{c} B=\frac{E_{\text{irrev}}-E_{\text{rev}}}{8}, \end{array}$$

where *E*_rev_ and *E*_irrev_ are the thresholds for electroporation (kV/m) and irreversible electroporation (kV/m), respectively, *σ*_max_ represents the maximum electrical conductivity reached during the tissue electroporation (S/m), and *σ*_0_ is the basal (or initial) tissue electrical conductivity (S/m), which is measured with low amplitude pulses. The values for *σ*_0_ are often extrapolations from measures held at 10–100 Hz [6, 18, 20]. During the application of pulses intense enough to produce electroporation (i.e., above *E*_rev_), the tissue electrical conductivity varies as described by (2) [20]. Tissue electrical conductivity, as a function of the electric field, reaches a constant value, called *σ*_max_, inasmuch as the local electric field approaches *E*_irrev_ (Figure 2). The postelectroporation conductivity (*σ*_max_) values are usually estimated through mathematical modeling with data from ex vivo or *in vivo* experiments [7, 20]. In this work, the tissues were characterized by using the values from Table 1 in (2)–(4).

#### 2.2.2. Numerical Modeling

The electric field distributions of the tissues were computed through the finite element method simulations with COMSOL Multiphysics (v5.0, COMSOL AB, Sweden). The software was run on a personal computer (Intel Core i5-2500, 3 GHz CPU, 4 GB RAM) with a Windows 7 (x64, Microsoft, Inc., USA) operating system.

The geometry presented (Figure 1) was automatically divided into a mesh of ∼162 thousand tetrahedral elements forming the calculation domains. The electric field distribution developed by the applied electric potential on the tissues is governed by Laplace's equation (3), and it was solved for static electric currents as follows:

(5) $$\begin{array}{c} -\nabla \cdot \left(\sigma \cdot \nabla V\right)=0, \end{array}$$

where *σ* and *V* are tissue electric conductivity (S/m) and electric potential (*V*), respectively. The considerations for boundary conditions were that all external surfaces are insulated (Neumann's boundary condition). For the contact tissue electrodes, Dirichlet's boundary condition, considering a constant potential on the surface of all the electrodes, was applied.

### 2.3. Treatment Planning Simulation

**Figure 3:**
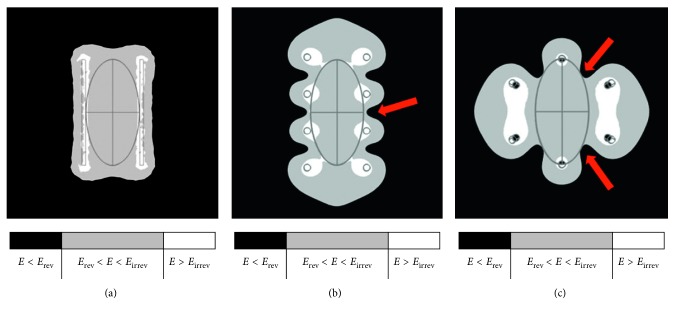
Typical options for the application of electrochemotherapy using (a) parallel plates, type I (b) parallel needles, type II, or (c) hexagonal needles, type III. Simulations show the electric field distribution for the minimum and sufficient applied voltage for electroporation (a) 75 kV/m, (b) 110 kV/m, and (c) 127.5 kV/m. The local electric field is in black and is insufficient for electroporation, the electroporated area is on gray, and the irreversible electroporated areas are in white. The arrows indicate electric field indentation close to the tumor edges.

**Figure 4 fig4:**
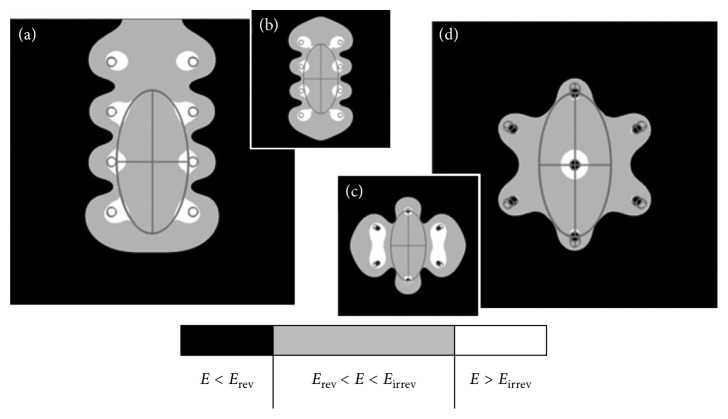
Electric field distribution for (a) type II electrode replacement in the *X*-axis and (d) type III electrode with an extra needle added. Type II (b) and type III (c) were taken from Figure 1 for comparison purposes. The gray areas represent electroporated regions, while black and white mean the magnitude of the local electric field is under or over the respective thresholds.

**Figure 5:**
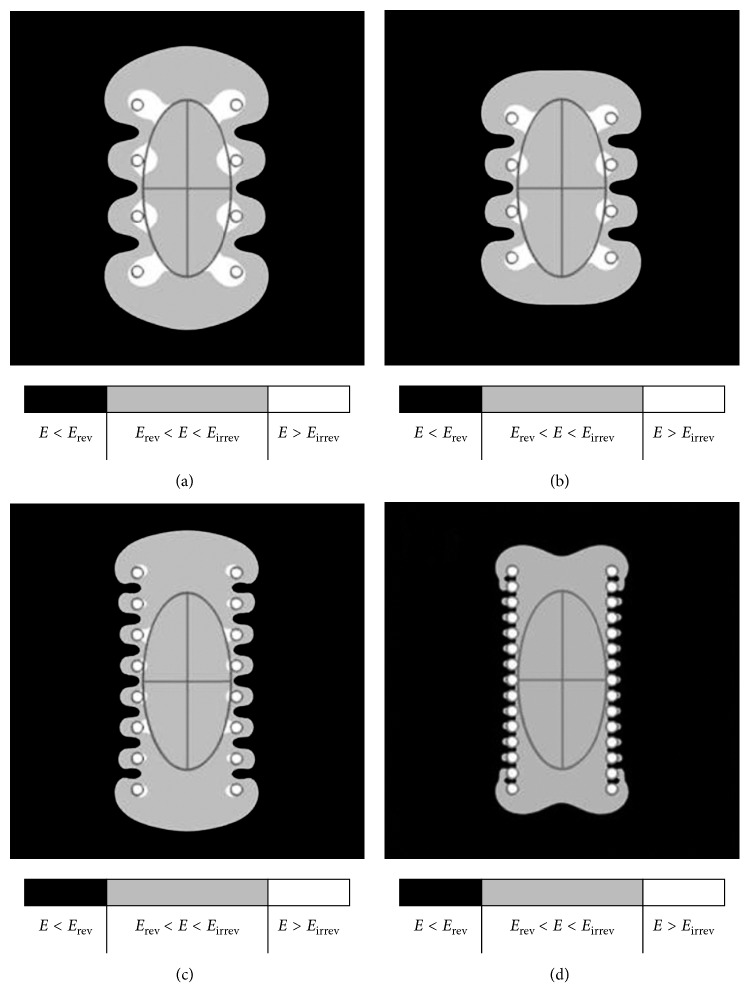
Decreasing the distance between needles in the same column (same polarity) reduces the minimum required voltage. The sequence of panels also shows a reduction of the irreversible electroporated area in comparison with (a). The distance between needles was reduced to (a) 90%, (b) 75%, (c) 50%, and (d) 25% of the original 7 mm. The initial 1100 V minimum required voltage was reduced by approximately (a) 2%, (b) 14%, (c) 30%, and (d) 50%.

The treatment effectiveness depends on the capacity of the system to produce a local electric field high enough to open pores around the entire tumor [8–10, 12]. In the simulation environment, the local electric field indicates whether the electroporation of the tumor cells is theoretically viable. Whenever the local electric field was in the range of 35 kV/m–100 kV/m [18], it was assumed that the pores were open, allowing the influx of the chemotherapy drugs. In regions where the local electric field is lower than 35 kV/m, the induced transmembrane voltage is not considered sufficient to trigger pore formation [5, 18]. In other words, there is a loss of effectiveness when regions of the target tissue (i.e., tumor cells) are exposed to a local electric field lower than 35 kV/m during an electrochemotherapy session. The regions with no pore formation are shown in black in the results (Figures 3 [Fig fig4]–5). Irreversible electroporation areas are represented in white, and they indicate that the cells in these areas lost the ability to reseal. Irreversible electroporated cells may also die but not due to the action of the chemotherapy drugs [2, 11]; therefore, an investigation into the death of these cells is beyond the scope of this study. A manual optimization process was carried out, aiming to maximize the region inside the range 35 kV/m and 100 kV/m and to minimize the tumor portions under or overexposed. During this process, the model was rerun several times for different inputs, and the outputs were evaluated.

A minimization process was run to determine the sufficient and necessary applied electric field to electroporate all target cells with each arrangement. The applied voltage was minimized through several simulations. The initial point was 130 kV/m, and this value was decreased in the following experiments. The minimization process was carried out to determine the electric field sufficient to create a local electric field and high enough to electroporate the cells. The tridimensional structure was cut into slices for the three spatial planes (*ZX*, *XY*, and *YZ*). In these slices (e.g., Figures 3 [Fig fig4]–5), the local electric field was considered “sufficient” when higher than 35 kV/m (the electroporation threshold). This process was performed for each tested arrangement of needles, and the minimum values obtained are listed in Table 2. These minimum values are used as classification parameters for the robustness of the arrangements. The classification is based on how far each minimum value is from the starting point (i.e., 130 kV/m).

## 3. Results

The electric field distribution for the three main types of electrode (Figure 3) showed that the adequate type I affects the healthy tissue less than the other types. The tested types were the parallel plates (Figure 3(a)), parallel needles (Figure 3(b)), and hexagonal needles (Figure 3(c)). The regions in black represent the absence of electric field, while those in white represent the extrapolation of the local electric field. The minimum and sufficient values of the applied electric field found for these electrodes were 75 kV/m (Figure 3(a)), 110 kV/m (Figure 3(b)), and 127.5 kV/m (Figure 3(c)). Type I (a) presented the least electroporated area (gray) among the healthy tissues, that is, out of the ellipse. Type III (c) required the highest electric field due to the large indentation in the corners (indicated by the arrows), resulting in large areas of irreversibility (white).

Variations from the basic types resulted in reduced values of minimum and sufficient applied voltage for electroporation.  Figure 4 shows the electric field distribution when the electrode was moved from the central position (Figure 4) and when a needle pair was placed around the tumor's largest diameter in the *Y*-axis. In this last scenario, no other needle is closer to the tumor than the pair on the largest diameter.  Figure 4 shows that the type III electrode (Figure 4) had one extra needle inserted in the center. The centered needle's polarity was the opposite of the others, and a large area of irreversible electroporation was observed in the center.

To better elucidate how the distance between needles with the same polarity changed the electrical field distribution, several in silico experiments were run. The original distance between needles with the same polarity in the same column (*d*_N_) was 7 mm. Four variations in *d*_N_ were tested and are presented in Figure 4. The *d*_N_ was reduced to 90% (Figure 5(a)), 75% (Figure 5(b)), 50% (Figure 5(c)), and 25% (Figure 5(d)). The number of needles was increased to minimize the spreading effect at the borders.

The necessary and sufficient values of the applied voltage for the in silico experiments discussed in this work are listed in Table 2, with the best result seen in the last line. The presented values were sufficient to electroporate all the cells in the region between electrodes. The first column indicates the tested arrangements as plates, needles, and their variations. The second and third columns show the obtained values and the percentages from type II as a reference, respectively. Type II electrodes are most commonly employed for cutaneous tumors [3]. The last column indicates which figure represents each result.

Graphical visualization for the tested arrangement of electrodes is presented in the first column. The positive electrodes are gray and the ground electrodes are black. Three tridimensional models can be seen in Figure 1.

## 4. Discussion

In this age of electronics, the health sciences have received contributions from many fields, such as bioinformatics, magnetic resonance imaging, and robotic hands for surgeries. Treatment planning is a powerful tool during the preoperative stage that allows clinicians to predict eventual complications or loss of effectiveness [10, 21, 22]. Thanks to fast and powerful modern processors, real-time simulations may even be run at the same time of surgery in case recalculations are needed [21, 22]. Electrochemotherapy is an anticancer treatment approach kept allied to numerical simulations since its early days [15, 20]. In this work, we use a simple study case to demonstrate a treatment planning procedure based on numerical simulation. The presented results allowed us to highlight the loss of potential treatment effectiveness due to the electric field indentation between needles with the same polarity.

The ellipsoidal tumor presented in Figure 1 was modeled considering specific electrical and geometrical characteristics (Figures 1 and 1), and the arrangements of different electrodes were evaluated. Once the target tumor tissue was modeled, several simulations were run considering variations in, for instance, the electrodes' type, position, arrangement, and polarization. We followed the suggestions by the European Standard Procedure for Electrochemotherapy (ESOPE), which are based on the number and volume of the tumors [1]. For large (>8 mm) superficial tumors, ESOPE suggests using type I or III electrodes [1]. Based on the in silico results presented for the three commonly used electrode types (Figure 3), all electrode types allowed the treatment of the tumor under study. This means, for this tumor, all three electrodes were able to generate a local electric field sufficient to trigger electroporation. However, practicality and robustness are also important considerations in electrode choice. The type I electrode was shown to be the most robust because, even if the voltage source cannot supply the appropriate voltage, this type provides an effective treatment with 750 V. However, type I is also the electrode that drains the highest current from the source [15], especially when conductive gels are applied to increase the electric field's homogeneity [8, 11, 12].

High current peaks are the main reason for voltage drops, which are usually related to liquid accumulation in the tumor surroundings due to bleeding or suppuration. In addition to voltage drops, type I electrodes may be more difficult to handle than type II and III. During the early days of electrochemotherapy, the plates used to be attached to calipers for an easier measurement of the distance and the subsequent calculation of the required voltage to be applied [23]. After a few years, predefined electrode plates with fixed distances were commercialized and became widely used [1, 3, 16], skipping the necessity to recalculate the applied voltage for each repositioning. However, the ability to change the position of the plates to squeeze the tumor had already been demonstrated [16]. In this sense, the use of type I electrodes seems to be restrained to small tumors in superficial and soft tissues, which can be accommodated between the plates.

Type II electrode replacement reduced the minimum and sufficient voltage. In one of our experiments, where the position of the type II electrode was changed along the *X*-axis (Figure 4), a 7% decrease in the needed voltage to cover the tumor was observed. This was the first indication of the importance of the field indentation between needles with the same polarity. This result is important because it revealed the importance of a lateral safe margin, which was exploited in the following experiments.

When an extra needle was inserted into the center of the type III electrode (Figure 4), the electric field had a different distribution. Such an arrangement has been used for ECT in some cases [4, 23, 24], and it is also considered a good option for commuted systems (at least two different polarization steps for the needles) [24]. For a single polarization scenario, Figure 4 shows that the electroporated area is less irreversible in healthy tissue, but a large one around the center needle is observed (only one with the opposite polarization from the others). This electrode also presents a significant reduction (27%) of the sufficient and necessary voltage for treatment. However, this structural modification of the type III electrode might not be available to all physicians and veterinarians.

The spacing between needles is an important parameter for increasing the effective area, but based on the presented experiments, it was observed that reducing the space between needles with the same polarity in the type II electrode decreased the minimum required voltage and shrank the electric field indentation (Figure 5). These results also imply the need to structurally modify the commercial electrodes; however, there are extrapolations to show the electric field indentation in the region between needles with the same polarity. Independent of the space between the needles in the same row (*d*_N_), the applied electric field should produce the same effect in the tumor under treatment, as the electric field depends on the distance between rows (*E* = *V*/*d*_R_). However, we observed that the applied field could be continually decreased for type II electrodes with a smaller distance between the same polarity needles (<*d*_N_). The sufficient and necessary voltage is lower for smaller values of *d*_N_ (Table 2); therefore, 0.25∗type II is considered the most robust electrode. Based on these results, we highlight the impact of the field indentation between needles with the same polarity. For clinical use, needle electrodes are potentially vulnerable to losses in treatment effectiveness due to voltage drops, especially in cases where the tumor boundaries are close to the needles. This finding is contrary to what has been suggested for plate electrodes, where the tumor should be squeezed between the electrodes [16]. Although *in vitro* and *in vivo* experiments are still needed, our conclusion seems to be corroborated by previous studies based on genetic algorithms aiming for the most suitable distance between needles [14]. This conclusion is especially important because type II electrodes have been the most commonly employed in clinics [3]. At the same time, complete responses have been achieved in the clinic less often for large tumors than for small tumors (<3 cm) [3]. As large tumors require multiple pulse applications [1], overlapping applications to reduce blank areas are likely to increase treatment effectiveness. To the best of our knowledge, this is the first study to observe the importance of keeping a safe margin between the tumor and needle electrodes.

## 5. Conclusion

In silico experiments are a powerful approach to confirm well-understood concepts before or in parallel to *in vitro* and *in vivo* studies. Although the conclusions of this study still must be translated into *in vitro* and *in vivo* experiments, we showed that fundamental issues like a safe margin and effectiveness loss can be revealed using a validated numerical model. Even though type II electrodes are the most commonly used by practitioners [3], previous studies have pointed out the limitations of this treatment for large tumors. For example, the top regions of large tumors might not be electroporated without conductive gels [2, 11, 12]. The literature has also considered the depth of the tumor and the depth of needle insertion needed to avoid nonelectroporated areas at the tumor's bottom [8]. We presented numerical simulations that indicate the importance of considering the electric field indentation to make the treatment as effective as possible.

## Figures and Tables

**Table 1:** Tissue electrical parameters [17].

Tissue	*σ*_0_ (S/m)	*σ*_max_/*σ*_0_	*E*_rev_ (kV/m)	*E*_irrev_ (kV/m)
SC + epidermis	0.008	100	40	120
Dermis	0.250	4	30	120
Muscle	0.135	2.5	20	80
Tumor	0.300	2.5	40	80

**Table 2:** Necessary and sufficient voltage for electroporation.

	Arrangement	Voltage (kV)	∆%	Figure
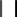	Type I	75	−31.8	3
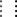	Type II	110	As reference	3
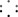	Type III	127.5	15.9	3
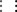	Type II 0X-moved	102.5	−7	4
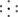	Type III + central needle	80	−27	4
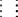	1.10∗type II	122.5	11.4	Not presented
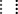	0.90∗type II	107.5	−2.3	5
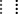	0.75∗type II	95	−13.6	5
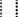	0.50∗type II	77.5	−29.5	5
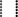	0.25∗type II	55	−50	5

## Data Availability

The data used to support the findings of this study are available from the corresponding author upon request.

## References

[1] Mir L. M., Gehl J., Sersa G. (2006). Standard operating procedures of the electrochemotherapy: instructions for the use of bleomycin or cisplatin administered either systemically or locally and electric pulses delivered by the Cliniporator by means of invasive or non-invasive electrodes. *European Journal of Cancer Supplements*.

[2] Rubinsky B. (2010). Irreversible electroporation. *Series in Biomedical Engineering*.

[3] Matthiessen L. W., Chalmers R. L., Sainsbury D. C. G. (2011). Management of cutaneous metastases using electrochemotherapy. *Acta Oncologica*.

[4] Cen C., Chen X. (2017). The electrode modality development in pulsed electric field treatment facilitates biocellular mechanism study and improves cancer ablation efficacy. *Journal of Healthcare Engineering*.

[5] Ho S. Y., Mittal G. S. (1996). Electroporation of cell membranes: a review. *Critical Reviews in Biotechnology*.

[6] Gabriel C., Gabriel S., Corthout E. (1996). The dielectric properties of biological tissues: I. Literature survey. *Physics in Medicine and Biology*.

[7] Suzuki D. O. H., Berkenbrock J. A., Frederico M. J. S., Silva F. R. M. B., Rangel M. M. M. (2018). Oral mucosa model for electrochemotherapy treatment of dog mouth cancer: ex vivo, in silico, and in vivo experiments. *Artificial Organs*.

[8] Miklavcic D., Corovic S., Pucihar G., Pavselj N. (2006). Importance of tumour coverage by sufficiently high local electric field for effective electrochemotherapy. *European Journal of Cancer Supplements*.

[9] Suzuki D. O. H., Anselmo J., de Oliveira K. D. (2015). Numerical model of dog mast cell tumor treated by electrochemotherapy. *Artificial Organs*.

[10] Pavliha D., Kos B., Županič A., Marčan M., Serša G., Miklavčič D. (2012). Patient-specific treatment planning of electrochemotherapy: procedure design and possible pitfalls. *Bioelectrochemistry*.

[11] Ivorra A., Al-Sakere B., Rubinsky B., Mir L. M. (2008). Use of conductive gels for electric field homogenization increases the antitumor efficacy of electroporation therapies. *Physics in Medicine and Biology*.

[12] Suzuki D. O. H., Marques C. M. G., Rangel M. M. M. (2016). Conductive gel increases the small tumor treatment with electrochemotherapy using needle electrodes. *Artificial Organs*.

[13] Suzuki D. O. H., Berkenbrock J. A., de Oliveira K. D., Freytag J. O., Rangel M. M. M. (2017). Novel application for electrochemotherapy: Immersion of nasal cavity in dog. *Artificial Organs*.

[14] Corovic S., Zupanic A., Miklavcic D. (2008). Numerical modeling and optimization of electric field distribution in subcutaneous tumor treated with electrochemotherapy using needle electrodes. *IEEE Transactions on Plasma Science*.

[15] Brandisky K., Daskalov I. (1999). Electrical field and current distributions in electrochemotherapy. *Bioelectrochemistry and Bioenergetics*.

[16] Corovic S., Al Sakere B., Haddad V., Miklavcic D., Mir L. M. (2008). Importance of contact surface between electrodes and treated tissue in electrochemotherapy. *Technology in Cancer Research and Treatment*.

[17] Corovic S., Lackovic I., Sustaric P., Sustar T., Rodic T., Miklavcic D. (2013). Modeling of electric field distribution in tissues during electroporation. *BioMedical Engineering OnLine*.

[18] Lacković I., Magjarević R., Miklavčič D. (2010). Incorporating electroporation-related conductivity changes into models for the calculation of the electric field distribution in tissue. *IFMBE Proceedings*.

[19] Misdorp W. (2004). Mast cells and canine mast cell tumours: a review. *Veterinary Quarterly*.

[20] Miklavcic D., Sel D., Cukjati D., Batiuskaite D., Slivnik T., Mir L. M. Sequential finite element model of tissue electropermeabilisation.

[21] Cukjati D., Batiuskaite D., André F., Miklavčič D., Mir L. M. (2007). Real time electroporation control for accurate and safe in vivo non-viral gene therapy. *Bioelectrochemistry*.

[22] Sadeghi Naini A., Patel R. V., Samani A. (2010). CT-enhanced ultrasound image of a totally deflated lung for image-guided minimally invasive tumor ablative procedures. *IEEE Transactions on Biomedical Engineering*.

[23] Heller R., Jaroszeski M. J., Reintgen D. S. (1998). Treatment of cutaneous and subcutaneous tumors with electrochemotherapy using intralesional bleomycin. *Cancer*.

[24] Rebersek M., Corovic S., Sersa G., Miklavcic D. (2008). Electrode commutation sequence for honeycomb arrangement of electrodes in electrochemotherapy and corresponding electric field distribution. *Bioelectrochemistry*.

